# Institutional factors associated with the incidence rates of central line-associated bloodstream infection in California community hospitals

**DOI:** 10.1371/journal.pone.0274436

**Published:** 2022-09-30

**Authors:** Ella Calixta Nelson, Chia-Hui Wang, Garry Huang, Nai-Wen Kuo

**Affiliations:** 1 School of Health Care Administration, College of Management, Taipei Medical University, Taipei City, Taiwan; 2 Medial Health, New York City, NY United States of America; 3 Department of Real Estate and Built Environment, National Taipei University, New Taipei City, Taiwan; Syreon Research Institute, HUNGARY

## Abstract

Central line-associated bloodstream infections are frequent, deadly, costly, and preventable. The study aimed to explore how some hospital-related characteristics were associated with incidence rates of central line-associated bloodstream infections reported by community hospitals in California from January to December 2019. This retrospective, cross-sectional study used combined data from records submitted to the California Department of Public Health, California Open Data Portal, the California Health and Human Services Open Data Portal, and the American Hospital Directory by community hospitals in California with central line-associated bloodstream infections in 2019. Results showed that CLABSIs are significantly associated with bed capacity, health care system affiliation, ownership, and hospital accreditation status (*p* < 0.0001). CLABSI remains a relevant threat to patient safety and quality of care, even more so in the community hospital setting. Understanding if a relationship exists between institutional factors and CLABSI rates might better prepare leaders in healthcare organizations to reduce HAIs.

## Introduction

### Background

As one of the most common sources of preventable harm to patients, healthcare-associated infections (HAIs) are significant threats to patient safety [1,2]. Infections represent a severe challenge in modern medicine and are one of the most accurate indicators of the quality of patient care [3]. The United States Centers for Disease Control and Prevention (CDC) defines an HAI as “a localized or systemic condition resulting from an adverse reaction to the presence of an infectious agent(s) or its toxin(s)” [4]. Essentially, HAIs are infections patients can get while undergoing medical care in a health care institution. Central line-associated bloodstream infection (CLABSI) is just one of the major types of HAIs. A CLABSI is a primary bloodstream infection (BSI) in a patient with a central line for more than two consecutive calendar days before the development of the BSI and is not bloodstream-related to an infection at another site [5].

Intravascular catheters are indispensable for modern health care [6], as seen by their diverse range of indications, including drug and blood administration and blood sample collection. However, their use may have a risk of BSI associated with microorganisms that colonize the outer surface of the device or the fluid path when inserting the device and an infection that ensues during use [7].

Interestingly, of all the HAIs, CLABSIs are associated with a high-cost burden [8], which carries attributable mortality of 12%–25% [9]. As exemplified by a recent publication, we should note that CLABSI is the most common life-threatening complication of central venous catheters (CVCs). CLABSIs were identified with more than 28,000 deaths yearly and cost over $2 billion [8]. Extensive research has demonstrated that CLABSIs are linked with notable augments in the length of hospital stay and, ultimately, increased patient morbidity [10–12]. Moreover, a report estimated that the annual cost of caring for patients with CLABSIs is between $60 million and $460 million [13]. Yet, these numbers do not mirror the decline in productivity and other less quantifiable human and economic costs related to a severe HAI [14].

### Hospital characteristics related to quality of care: CLABSI

Roughly 5% - 10% of acute care hospital patients in the US will present with one or more infections, an unfavorable situation occurring in about 2 million patients, causing 90,000 deaths and likely cost $4.5 billion to $5.7 billion [15]. Furthermore, out of every 100 hospitalized patients, seven patients in progressive countries and ten patients in emerging countries acquire an HAI [16], making HAIs a challenge of present-day medicine [17] and an essential and accurate indicator of quality of care, adverse events, and patient safety issues [18]. An effective set of indicators provides adequate information to describe and assess the care offered to the patient efficiently; further, it provides precise and easily comparable results [19]. Following this, it is reasonable to associate HAIs, particularly CLABSIs, with various hospital characteristics. However, there is limited information regarding the extent to which specific hospital characteristics can influence the differences in the rates of these adverse events [20].

### California hospitals and health perspective

California is the most populous state in the USA, with a 39.2 million population, accounting for 11.8% of the total USA population. It has an area of about 163,696 square miles (423,970 km2) and is the 3rd largest state in the US, making California an important study target [21].

California’s general acute care hospitals are categorized into four major categories consistent with the National Healthcare Safety Network (NHSN) categories based on risk adjustments. These include Acute Care Hospital, Long-Term Acute Care Hospital, Critical Access Hospital, and Rehabilitation Hospital or Unit. From this, hospitals are further categorized based on the type of facilities, such as Major Teaching, Paediatric; Community; Long-Term Acute Care; Critical Access; Free-Standing Rehabilitation, and Rehabilitation Unit. A community hospital (non-federal acute care) is a California Department of Public Health (CDPH) classification for hospitals not listed as a major teaching, long-term acute care, critical access, pediatric, or rehabilitation. Community hospitals are further categorized by the number of active beds as reported by the hospital in the NHSN Annual Survey [22].

### Significance and objectives of this study

Central lines are abundantly utilized in critically-ill patients [23]. Notably, CLABSI is a prime cause of preventable HAIs, resulting in more extended hospital stays, increased hospital costs, and marked mortality [24,25]. California has a state mandate to publicly report at least one HAI to NHSN [26]. California hospitals announced no significant change in CLABSIs between 2013 and 2014 [27]. Hence, this study explores how some hospital-related characteristics are associated with the incidence rates of CLABSI reported in California community hospitals from January 2019 to December 2019.

## Materials and methods

### Conceptual framework

**Fig 1:**
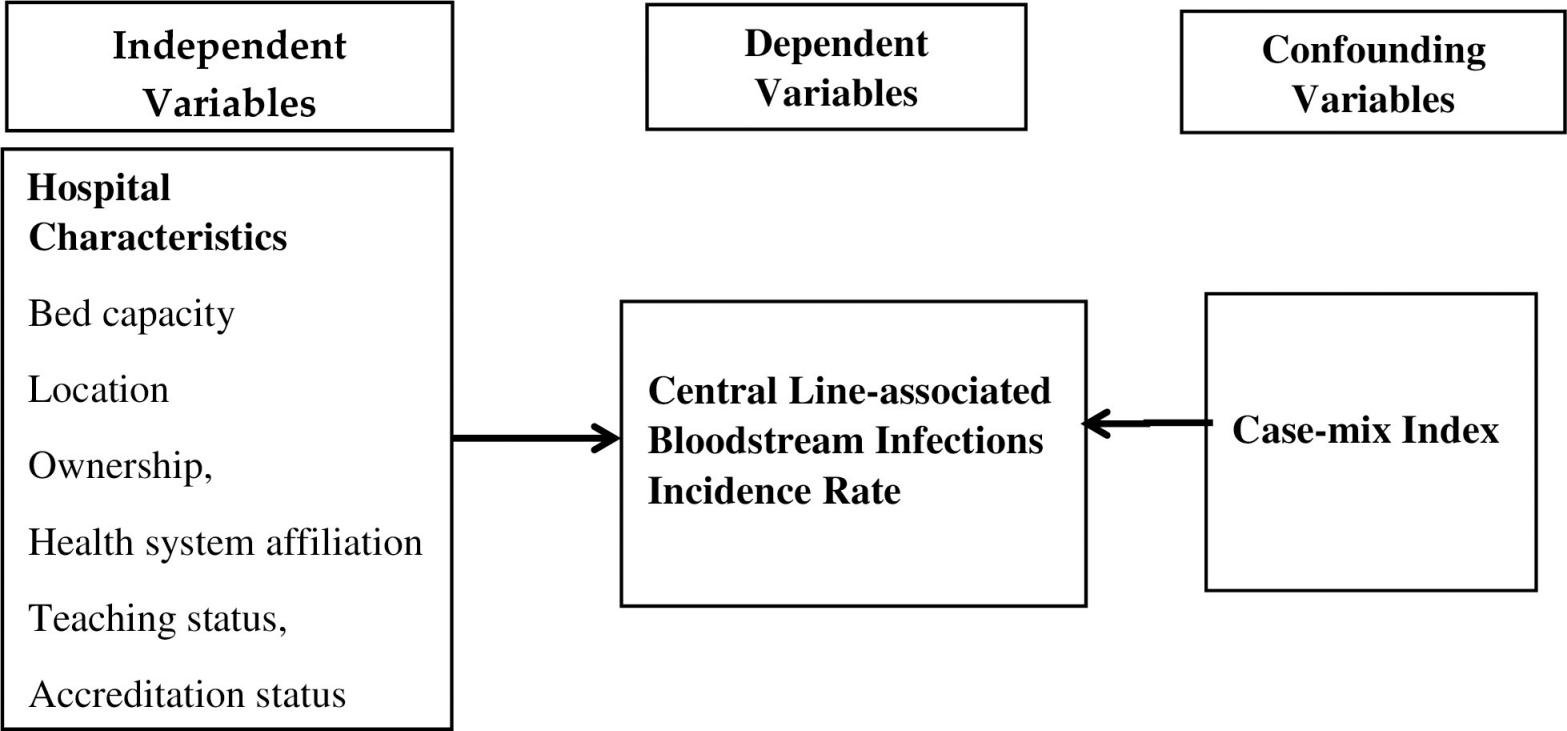
Conceptual framework.

Fig 1 presents the conceptual framework of this study. The dependent variable here is the central line-associated bloodstream infection incidence rate. Independent variables include bed capacity, location, ownership, health system affiliation, teaching status, and hospital accreditation status, while the case-mix index serves as the confounding variable.

### Operational definitions of variables

Table 1 describes the variables used and their operational definitions in this study. This study is a retrospective, cross-sectional and non-experimental research to examine associations between various hospital-related characteristics and the incidence of central line-associated bloodstream infection in California community hospitals.

**Table 1:** Operational definitions of variables in the analyses.

Variable	Operational Definition		Scale	Type of Variable
**Dependent Variable**				
CLABSI incidence rate	the number of CLABSI cases divided by the number of central line days and multiplied by 1000.	Per 1 000 central line days		Discrete
**Independent Variable**				
Bed capacity	Number of licensed beds		Under 125; 125–249; 250 and above	Categorical (ordinal)
Location	Location of the facility		Urban area; rural area	Categorical (nominal)
Ownership	Owner of the health care facility		Government; Non-profit; For-profit	Categorical (nominal)
Health system affiliation	Close tie of a health care facility to a larger organization		Affiliated; non-affiliated	Categorical (nominal)
Teaching status	Presence of a teaching program for medical students and/or a post-graduate medical training program		Teaching; Non-teaching	Categorical (nominal)
Hospital Accreditation	Hospitals that meet the Joint Commission International standards and evaluation methods		Accredited; non-accredited	Categorical (nominal)
**Cofounding Variable**				
Case-mix index	The average relative diagnosis-related group (DRG) weight of a hospital’s inpatient discharges		Payment rate	Discrete

The researcher hypothesized that there are statistically significant differences in reported CLABSI rates based on bed capacity, hospital location, ownership, health system affiliation, teaching status, and hospital accreditation status.

### Study population and data sources

This research included hospitals in California, USA, participating in mandatory NHSN, CMS, and CLABSI reporting. The study used pre-existing data from the California Department of Public Health, California Open Data Portal, the California Health and Human Services Open Data Portal, and the American Hospital Directory. The combined data sources provided 2019 clinical information for all California hospitals, hospital characteristics information of all California hospitals, and the 2019 fiscal year report. The merged data set contained information on 226 community hospitals for one year, from January 2019 to December 2019. All datasets are open to the public and are not patient identifiable. This research also excluded community hospitals that did not comply with the mandatory monthly submission of CLABSI rates for any given month in the study year. Therefore, there were 218 hospitals included in the analyses. This study only used de-identified data and large sample sizes available via public domains. Therefore, informed consent was non-applicable.

### Processing of data and statistical analyses

Firstly, descriptive statistics showed hospital characteristics. Due to the non-parametric nature of the data, we performed the Mann-Whitney *U* test and Kruskal-Wallis H test to determine the effect of hospital characteristics on CLABSI incidence rates. Additionally, we also adopted Pearson’s correlation analysis to probe the relationship between each of the independent variables and the dependent variable. Finally, given many zero-reported CLABSI episodes, a zero-inflated Poisson regression facilitated the analysis of the association between the independent and control variables with the dependent variable.

## Results

### Description of the hospital-related characteristics

Table 2 summarizes the hospital-related characteristics of the 218 participating California community hospitals and non-participating hospitals versus all 393 California acute hospitals in 2019. The licensed bed capacity of <125, 125–249, and ≥250 was (38.1%), (33.9%), and (28%), respectively, averaging 200 beds. Hospitals located in urban areas accounted for (92.7%). More than half (57.8%) of the hospitals were non-profits. Government and for-profit accounted for (11.9%) and (30.3%), respectively. California community hospitals affiliated with a health system were proportioned at (85.1%), whereas non-affiliated hospitals were at (14.9%). The majority of the hospitals (95.8%) provided teaching programs. Fully Joint Commission accredited hospitals account for 96.4%, compared with 3.6% of non-accredited hospitals. Table 2 further presents the results of the Chi-square tests. It shows no significant difference between sample hospitals and the population regarding bed capacity, location, ownership, system affiliation, and accreditation status. These two groups are, therefore, comparable except in their teaching status.

**Table 2:** Characteristics of participating, non-participating vs. all California hospitals.

*Hospital Characteristics*	(1) Participating hospitals (*N* = 218)	(2) Non-participating hospitals (*N* = 175)	(3) All California hospitals (*N* = 393)	*Ch-square value* *(p-*value*)* (1) vs. (2)	*Ch-square value* *(p-*value*)* (1) vs. (3)
**Bed capacity**					
Under 125	38.07% (83)	40.57% (71)	39.19% (154)	8.95 (0.011*)	2.5 (0.286)
–249	33.94% (74)	21.14% (37)	28.24% (111)		
250 and above	27.98% (61)	38.29% (67)	32.57% (128)		
**Location**					
Urban	92.67% (202)	83.43% (146)	88.55% (348)	8.16 (0.004*)	2.64 (0.104)
Rural	7.33% (16)	16.57% (29)	11.45% (45)		
**Ownership**					
Government	11.93% (26)	16.57% (29)	14.00% (55)		1.01 (0.603)
Non-Profit	57.80% (126)	58.29% (102)	58.01% (228)	2.41	
For-Profit	30.27% (66)	25.14% (44)	27.99% (110)	(0.299)	
**System affiliation**					
Affiliated	80.73% (176)	65.71% (115)	74.05% (291)	11.39 (0.001*)	3.4 (0.062)
Non-affiliated	19.27% (42)	34.29% (60)	25.95% (102)		
**Teaching status**					
Teaching	95.41% (208)	4.00% (7)	54.71% (215)	327.37 (*p <* .001*)	109.07 (*p <* .001*)
Non-teaching	4.59% (10)	96.00% (168)	45.29% (178)		
**Hospital Accreditation**					
Accredited	92.20% (201)	90.29% (158)	91.35% (359)	0.45 (0.502)	0.13 (0.715)
Non-accredited	7.80% (17)	9.71% (17)	8.65% (34)		

Main data sources: California Health and Human Services Open Data Portal- Hospital Annual Financial Data: https://data.chhs.ca.gov/dataset/hospital-annual-financial-data-selected-data-pivot-tables.

However, the distributions of hospital characteristics between participating and non-participating hospitals are significantly different in bed capacity, location, system affiliation, and teaching status, except for their ownership and accreditation status.

The data showed that 637 CLABSI episodes occurred within 1,176,561 central line days. The mean CLABSI rate per 1000 central line days was 1.09 with an *SD* of 6.99. Of the reporting hospitals, (35.8%) had a CLABSI rate of zero. The max number of cases reported by an individual facility was 50.

### Multivariate analysis

Table 3 reports the zero-inflated Poisson model results using the dependent, independent, and control variables. We adopted the following variables as the baseline of their respective categories: rural (location), government (ownership), non-affiliated (health system affiliation), non-teaching (teaching status), and non-accredited (hospital accreditation).

**Table 3:** The association between hospital characteristics and incidence rates of central line-associated bloodstream infection in California community hospitals.

Variable	Estimate	SE	*p*	95% CI.
Intercept	.20	1.10	.857	-1.96, 2.36
CMI	.81	.32	.010*	.19, 1.43
Bed capacity	-.00	.00	< .0001	-.01, -.00
Urban	1.11	.65	.087	.16, 2.38
Non-profit	-1.57	.18	< .0001	-1.93, -1.00
For-profit	-1.89	.23	< .0001	-2.34, -1.43
Health system affiliated	1.17	.79	< .0001	.83, 1.53
Teaching	.83	.00	.292	-.71, 2.37
Accredited	-1.99	.18	< .0001	-2.34, -1.64

*Note*. All significance tests are two-tailed

*p < .05.

According to the model in Table 3, a few hospital characteristics were statistically significant in predicting the occurrence of CLABSI. Bed capacity was a significant predictor of the incidence rates of CLABSI, suggesting that increased bed capacity decreased the predicted CLABSI rate (*p* < 0.0001). Compared with government-owned hospitals, non-profit and for-profit hospitals demonstrated significantly lower CLABSI rates (*p* < 0.0001). Compared to non-system affiliated hospitals, system-affiliated hospitals showed significantly higher CLABSI incidence rates (*p* < .0001). Accredited hospitals were associated with significantly lower CLABSI incidence rates (*p* < .0001). This finding was indicative of non-accredited hospitals experiencing higher CLABSI rates. The association between the CMI scores and the incidence of CLABSI rates is also statistically significant (*p* = 0.010).

There are statistically significant differences in reported CLABSI rates based on bed capacity, ownership, health system affiliation, and hospital accreditation status. However, there is no statistically significant difference in reported CLABSI rates based on hospital location and teaching status.

## Discussion

### Research findings

Institutional factors for CLABSI are dynamic and complex in any given hospital setting. In this retrospective study, bed capacity, location, ownership, health system affiliation, teaching status, and accreditation status were significantly correlated with CLABSI. There were 637 CLABSI cases reported during the study period, and the average CLABSI rate was 1.09.

The results found from this study supported the hypothesis that there is a statistically significant difference in CLABSI rates based on bed capacity. There was substantial evidence to suggest hospitals with increased bed capacity result in decreased incidence rates of CLABSI. Although not investigated independently, one author identified a correlation between hospital type and size with HAIs, CAUTI, and CLABSI [28]. Another found a CLABSI infection ratio in ICUs was 18% more in hospitals with 400 or more beds than in hospitals with less than 200 beds [29]. This result is inconsistent with other studies [3,30].

This study did not demonstrate any significant relationships between location and CLABSI rates. The existing literature argues that rural hospitals usually lack infectious disease specialists and resources for infection control [31] which could ultimately increase infection rates. The rural facility may not be able to meet or handle the demands. Subsequently, rural hospitals are difficult to maintain a high level of skill and training to deliver high-quality care in their infection prevention programs [32]. Its urban counterparts, however, do not face such challenges. Most rural hospitals are government-owned or fall under some other non-profit classification [33], and they may inherently experience similar CLABSI rates.

The association between ownership and CLABSI incidence rate was statistically significant for non-profit and for-profit hospitals compared with government hospitals. Similarly, previous work has demonstrated an association between type of hospital ownership and CLABSIs and other HAIs [34,35]. However, this finding was inconsistent with a study done on hospitals in 41 US states, which concluded that ownership type was not associated with rates of any HAI [36].

There is a statistically significant difference in reported CLABSI rates based on health system affiliation. More so, research has found that medical school affiliation is an important factor for device-associated infection rates (central lines) and percentile distributions in medical ICUs and surgical ICUs [37].

Education is vital for CLABSI reduction. Surprisingly, there is no significant finding in this study. This result may likely be due to the extensive work on education-based programs focused on preventing CLABSI [38]. Ultimately, the CLABSI rate widely differs between health care institutions and mirrors the infection protection, prevention, and control practices or measures [39].

This study found hospital accreditation status is also associated with CLABSI incidence rates. The accredited community hospitals in this study showed significantly lower CLABSI rates. A considerable amount of data suggest that accredited hospitals are more inclined to adhere to evidence-based guidelines [40]. Likewise, another study also supports that accredited hospitals must adopt policies and practices in line with evidence-based practices to mitigate the risk of CLABSIs [41].

Lastly, results showed that the CMI score is significantly associated with CLABSI incidence rates. This result is reasonable since central lines are abundantly utilized in critically-ill patients [23].

### Limitations and recommendations for further research

One of the significant limitations of this study is that this was a secondary/non-experimental analysis rather than a primary/experimental analysis. The data presented were solely from California community hospitals. This study may not be generalizable to institutions that do not match the general characteristics of hospitals of this study. Moreover, many health facilities in this study reported zero episodes of infections, so the researchers had to adjust to using the zero-inflated Poisson model for statistical analysis.

Additionally, due to the unavailability of the facility and patient-level data, this study could not calculate standardized infection ratios (SIRs) like the National Healthcare Safety Network (NHSN) does [42]. The SIRs adjust for various facility and patient-level factors contributing to HAI risk within each facility. The advantage of using SIRs is allowing more equal comparisons across hospitals. Furthermore, due to the secondary data nature, this study could not evaluate many other characteristics such as facility type based on medical specialty (e.g., oncology, surgical, etc.) and type of CLABSI (e.g., mucosal barrier injury, medical life support device-related, etc.).

There is an urgent need for future studies to identify specific hospital characteristics and practices to focus on prevention strategies in this understudied population to monitor the burden of CLABSIs and HAIs. For this reason, it is recommended that the association between nurse staffing ratio and CLABSI incidence rates in community hospitals be investigated. Nurses are pivotal in CLABSI prevention, as they work closely with patients and perform most of the central line maintenance [43].

We suggest that further research uses multiple years of data (a longitudinal study approach) to evaluate infection trends better. It would be interesting to look into those facilities that reported zero episodes of CLABSI to establish whether the figures were related to under-reporting by the facility or simply due to a lack of resources and/or services. Likewise, a better in-depth understanding of hospital ownership’s drastic differences and role in hospital infections is necessary.

## Conclusion

CLABSI remains a relevant threat to patient safety and quality of care, even more so in the community hospital setting. Understanding if a relationship exists between institutional factors and reported CLABSI rates might better prepare leaders in health care organizations to reduce HAIs. This study provides a starting point for understanding the incidence of CLABSIs and the resulting burden in California community hospitals.

Although California’s HAI incidence continues to improve each year, it is not decreasing for all hospital infection types; thus, they are unlikely to meet the 2020 HAI reduction goals for CLABSI [44]. Therefore, California hospitals should enhance their HAI prevention efforts forward to improve patient safety. The results yielded from this study can be of use to hospital administrators/managers, physicians, and policymakers because it highlights the possible deficiencies represented by hospital factors that impact CLABSI incidence rates within community hospitals. It may also assist with strengthening the HAI surveillance and reporting systems in this understudied health care setting.
